# Vimentin is involved in regulation of mitochondrial motility and membrane potential by Rac1

**DOI:** 10.1242/bio.011874

**Published:** 2015-09-14

**Authors:** Elena A. Matveeva, Larisa S. Venkova, Ivan S. Chernoivanenko, Alexander A. Minin

**Affiliations:** Institute of Protein Research, Russian Academy of Sciences, Department of Cell Biology, Moscow 119988, Russia

**Keywords:** Cytoskeleton, Mitochondria, Vimentin intermediate filaments, Rac1

## Abstract

In this study we show that binding of mitochondria to vimentin intermediate filaments (VIF) is regulated by GTPase Rac1. The activation of Rac1 leads to a redoubling of mitochondrial motility in murine fibroblasts. Using double-mutants Rac1(G12V, F37L) and Rac1(G12V, Y40H) that are capable to activate different effectors of Rac1, we show that mitochondrial movements are regulated through PAK1 kinase. The involvement of PAK1 kinase is also confirmed by the fact that expression of its auto inhibitory domain (PID) blocks the effect of activated Rac1 on mitochondrial motility. The observed effect of Rac1 and PAK1 kinase on mitochondria depends on phosphorylation of the Ser-55 of vimentin. Besides the effect on motility Rac1 activation also decreases the mitochondrial membrane potential (MMP) which is detected by ∼20% drop of the fluorescence intensity of mitochondria stained with the potential sensitive dye TMRM. One of important consequences of the discovered regulation of MMP by Rac1 and PAK1 is a spatial differentiation of mitochondria in polarized fibroblasts: at the front of the cell they are less energized (by ∼25%) than at the rear part.

## INTRODUCTION

Mitochondria play one of the central roles in cell physiology, being not only the main energy source but also the regulatory hub of many cellular processes including apoptosis. The interaction of mitochondria with cytoskeleton as one of the crucial factors for their normal functions is in the limelight of many studies. First, the cytoskeleton ensures timely delivery and distribution of these organelles in cytoplasm. The transport of mitochondria provided by microtubules and actin microfilaments for their proper distribution in neurons was convincingly demonstrated by P. Hollenbeck and co-workers (Morris and Hollenbeck, 1995; Hollenbeck and Saxton, 2005). Several molecular motors that could be involved in this movement have been identified (Nangaku et al., 1994; Tanaka et al., 1998). Besides, there is substantial evidence that not only transport of mitochondria to the sites of high energy demand but also their retaining at these sites provided by cytoskeletal structures is important for their functions (Chada and Hollenbeck, 2003). Several mechanisms for ‘docking’ of mitochondria to microtubules (Leterrier et al., 1994), F-actin bundles (Minin et al., 2006), and intermediate filaments (IF) (Wagner et al., 2003; Winter et al., 2008; Nekrasova et al., 2011) have been proposed on the basis of video-microscopic, ultrastructural, and biochemical studies. However, many aspects of regulatory mechanisms that govern mitochondrial motility and their anchoring to various cytoskeleton structures are still unclear.

Last years an intensive attention was attracted to the IF as the scaffold structures for mitochondria because the dysfunctions of these organelles were observed in many cases as a result of IF disturbance. For example, mutations in desmin IF cause changes in the distribution and function of mitochondria in skeletal muscles and heart (Capetanaki, 2002), and the mutation in the neurofilament light chain that causes Charcot–Marie–Tooth disease results in the clustering of mitochondria in the cell bodies of neurons (Brownlees et al., 2002). There is an abnormal distribution of mitochondria in keratinocytes of patients with epidermolysis bullosa simplex, caused by mutations in keratins 5 and 14, (Uttam et al., 1996), and hepatocytes expressing mutant keratins 8 and 18 show enhanced susceptibility to apoptosis due to abnormalities in mitochondria (Gilbert et al., 2001). Morphological and functional changes in mitochondria have also been reported in vimentin-null fibroblasts (Tolstonog et al., 2001).

We have recently found that N-terminal part of vimentin molecule contains the sequence responsible for the interaction of vimentin IF with mitochondria (Nekrasova et al., 2011). This interaction causes not only the decrease of mitochondrial motility (Nekrasova et al., 2011) but also the elevation of mitochondrial membrane potential (MMP) (Chernoivanenko et al., 2015). Since VIF is likely to alter mitochondrial function it is reasonable to assume that their binding is under the regulatory control. According to our data the mitochondrial motility can be regulated by small GTPases of Rho family (Minin et al., 2006). Activated RhoA through its effector mDia1 inhibits the movements of mitochondria while activation of Rac1 has an opposite effect (Minin et al., 2006). In this study we demonstrate that Rac1-dependent increase of mitochondrial motility is explained by the disruption of their attachment to VIF. We show that Rac1 acts through its effector PAK1 kinase and causes the phosphorylation of vimentin on Ser-55 modifying the mitochondria-binding site. Consequently, released mitochondria acquire the higher motility and the reduced MMP.

**Abbreviations list**

IF: intermediate filament
VIF: vimentin intermediate filament
ATP: adenosine triphosphate
FBS: fetal bovine serum
MMP: mitochondrial membrane potential
TMRM: tetra-methyl-rhodamine-methyl ester
DMEM: Dulbecco's modified Eagle's medium
PAK1: p21-activated kinase
PID: PAK inhibition domain

## RESULTS

### Rac1 increases the motility of mitochondria

**Fig. 1.**
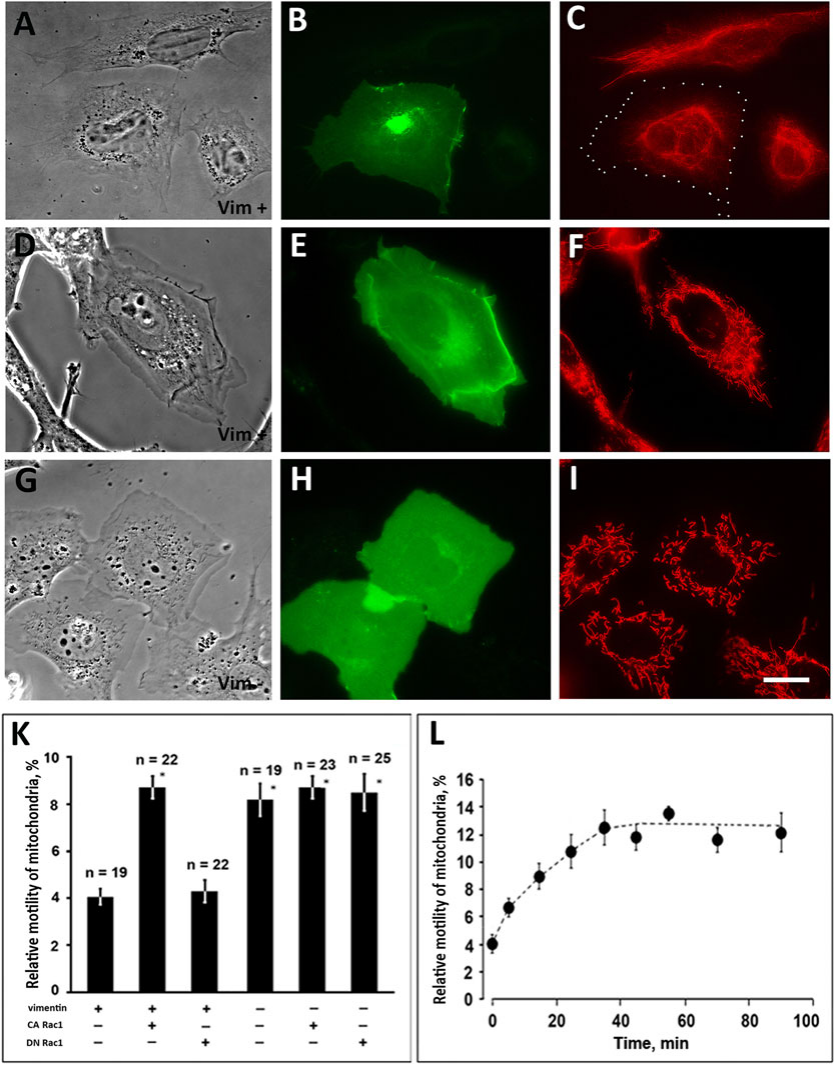
**Activated Rac1 increases mitochondria motility in vimentin-containing cells.** Cells MFT-6 (Vim+) (A-F) or MFT-16 (Vim−) (G- I) were transfected with plasmid encoding CA Rac1 [EGFP-Rac1(G12V)] and after 18 h were either fixed and immunostained for vimentin (C) or stained with Mitotracker Red to detect mitochondria in live cells (F,I). The transfected cells are revealed by GFP fluorescence (B,E,H). Panels A, D and G are phase contrast images of the cells shown in C, F and I, respectively. The margins of transfected cell in C are indicated by white dots. Scale bar, 10 μm. (K) Motility of mitochondria was analyzed in MFT-6 (Vim+) and MFT-16 (Vim−) cells, control or expressing Rac1 mutant indicated below and stained with Mitotracker Red. Values are mean percentage of movements exceeding 0.2 μm/s±s.e.m.; *n*=number of cells (approximately 500 movements in each cell). *Significantly different from +Vim/−Rac1 (*P*<0.01). (L) Cells MFT-6 (Vim+) were transfected with plasmid encoding mCherry-PA-Rac1 and after 18 h were stained with MitoTracker Deep Red FM to detect mitochondria in live cells. Motility of mitochondria was analyzed in transfected cells before and at indicated time points after irradiation of cells with blue light. Values are mean percentage of movements±s.e.m. as in G; 5−10 cells were taken for each time point (approximately 500 movements in each cell).

To study the effect of Rac1 on the interaction of mitochondria with VIF we expressed the constitutive active or dominant negative mutants of Rac1 fused to EGFP in murine vimentin-null MFT-16 cell line and the line MFT-6 containing VIF. Transfected cells expressing EGFP-Rac1(G12V) acquired a distinctive roundish shape with broad lamellipodia along entire perimeter. Such characteristic morphology was observed both in vimentin-containing MFT-6 (Fig. 1A,D) and vimentin-null MFT-16 cells (Fig. 1G). As it was shown earlier (Helfand et al., 2011) VIF networks retracted from the expanded lamellipodia in MFT-6 cells transfected with EGFP-Rac1(G12V) (Fig. 1C) and were redistributed to the cell center. The majority of mitochondria in MFT-6 cells were also localized in the vicinity of nuclei though some of them were distributed in lamellae (Fig. 1F).

Analysis of mitochondrial movements in transfected cells showed that Rac1(G12V) increased the motility of mitochondria in cells that contained VIF (MFT-6) while in vimentin-null cells (MFT-16) having a higher mitochondrial motility the expression of Rac1(G12V) did not cause additional increase of motility (Fig. 1K; supplementary materials Movies S1 and S2). The dominant negative mutant EGFP-Rac1(T17N) did not alter the motility of mitochondria in both cell lines (Fig. 1K). To confirm that the increased motility of mitochondria observed in cells containing VIF was a result of the activation of Rac1 rather than of an expression of foreign proteins we used a photo-activatable Rac1 (PA-Rac1) (Wu et al., 2011). This construct is inactive until the transfected cells are kept under red light and can be activated by irradiation with blue light. The data in Fig. 1L demonstrate that mitochondrial motility increased gradually after PA-Rac1 activation and reached its maximum in 30 min. It should be noted that the motility of mitochondria in MFT-6 cells transfected with PA-Rac1 reached the level that these organelles show in the absence of VIF in MFT-16 cells. So, activation of Rac1 removes an inhibitory effect of VIF on mitochondrial motility (Nekrasova et al., 2011) that might indicate their release from the constraint caused by their interaction with VIF.

**Fig. 2.**
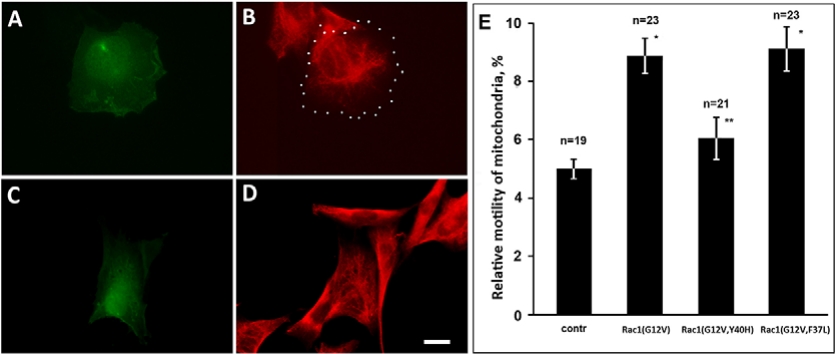
**Effects of Rac1(G12V), Rac1(G12V, Y40H), and Rac1(G12V, F37L) on mitochondria motility in vimentin-containing cells.** (A-D) Cells MFT-6 were transfected with plasmids encoding Rac1(G12V, Y40H) (A,B), and Rac1(G12V, F37L) (C,D) and after 18 h were fixed and immunostained for vimentin (B,D). The partial redistribution of VIF to cellular center is seen in cells transfected with Rac1(G12V, Y40H). The margins of transfected cell in B are indicated by white dots. Scale bar, 10 μm. (E) Movements of mitochondria were analyzed in control cells and cells expressing EGFP-Rac1(G12V), Rac1(G12V, Y40H), and Rac1(G12V, F37L) stained with MitoTracker Red. Values are mean percentage of movements exceeding 0.2 μm/s±s.e.m.; *n*=number of cells (approximately 500 movements in each cell). *Significantly different from control (*P*<0.01); **no significant difference from control (*P*>0.80).

Rac1 is involved in many regulatory processes and acts through different effector proteins (Westwick et al., 1997). To identify the effector pathway that mediates Rac1 regulation of mitochondrial behavior we expressed in cells containing VIF constitutively active effector loop mutants: Rac1(G12V, Y40H) defective in activating PAK1, and Rac1(G12V, F37L) which binds to PAK1 but not to other effectors (Joneson et al., 1996). As it was expected from previous studies an expression of these two double mutants had different impact on cell morphology. The cells expressing Rac1(G12V, Y40H) formed broad lamellae and had a roundish shape (Fig. 2A) similarly to those expressing Rac1(G12V) (Fig. 1A,B). Actin cytoskeleton in these cells was strongly altered: the stress fibers were not observed as it was shown earlier (Joneson et al., 1996; Meriane et al., 2000) (see supplementary material Fig. S1A,B). On the contrary, expression of Rac1(G12V, F37L) had no effect on cell morphology (Fig. 2C) or actin cytoskeleton (supplementary material Fig. S1A,B). The retraction of VIF caused by Rac1 activation was seen only in cells transfected with plasmid encoding Rac1(G12V, Y40H) (Fig. 2B) while in the cells expressing Rac1(G12V, F37L) VIF network remained unperturbed (Fig. 2D). However, the effect on mitochondrial motility was seen only in cells expressing Rac1(G12V, F37L). Fig. 2E shows that motility of mitochondria increased in cells transfected with plasmids encoding Rac1(G12V) and Rac1(G12V, F37L) while expression of Rac1(G12V, Y40H) had no such effect. There are three conclusions that could be made based on these data: (1) the rearrangements of actin cytoskeleton caused by expression of Rac1(G12V, Y40H) and apparent inhibition of RhoA do not affect motility of mitochondria; (2) increased motility of mitochondria is not a result of retraction of VIF observed in cells expressing Rac1(G12V, Y40H); (3) the effect of Rac1(G12V, F37L) expression on mitochondrial movements points to PAK1 kinase as a possible effector in this regulatory process.

### PAK1 is involved in the regulation of mitochondria motility by Rac1

**Fig. 3.**
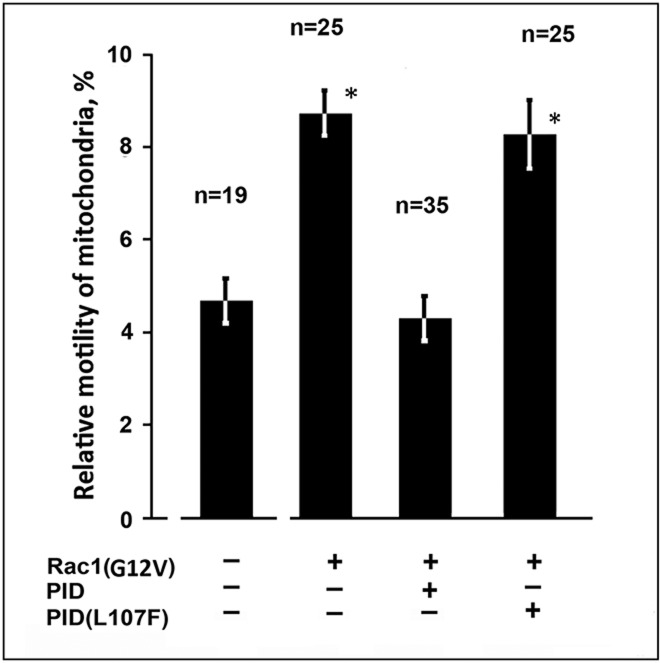
**Rac1 regulates mitochondria motility through PAK1 kinase.** Cells MFT-6 were transfected with plasmids encoding RFP-Rac1(G12V), EGFP-PID, and EGFP-PID(L107F). 18 h later cells were stained with MitoTracker Red and movements of mitochondria were analyzed. Values are mean percentage of movements exceeding 0.2 μm/s±s.e.m.; *n*=number of cells (approximately 500 movements in each cell). *Significantly different from control (*P*<0.01).

To elucidate the role of PAK1 kinase in regulation of mitochondrial motility by Rac1 we co-expressed auto-inhibitory domain PID of PAK1 (Zhao et al., 1998) fused to GFP together with RFP-Rac1(G12V) in cells containing VIF. The mutated form PID(L107F) which is unable to bind to catalytic domain of PAK1 was used as a control. Fig. 3 demonstrates that inhibition of PAK1 kinase suppressed the effect of Rac1(G12V) on mitochondrial motility while in the control it was elevated due to the expression of activated Rac1. Thus, activation of Rac1 causes the increase of mitochondrial motility through its effector PAK1 kinase in cells containing VIF. These data point to the possibility that VIF could be involved as a target of PAK1 kinase.

**Fig. 4.**
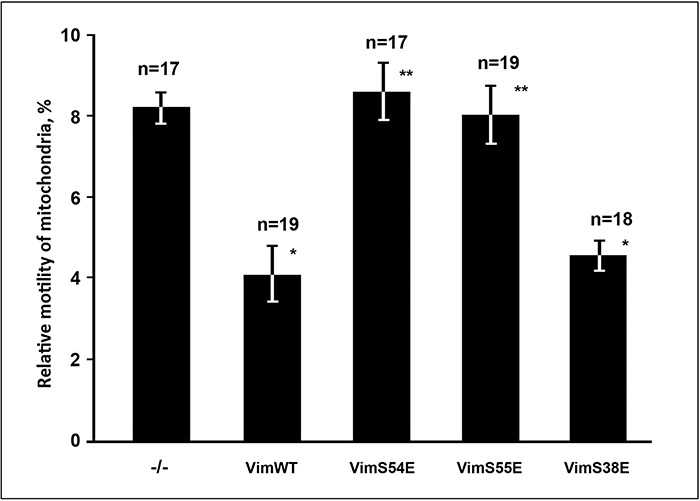
**Effects of vimentin mutations on mitochondria motility.** Cells MFT-16 were transfected with plasmid pIRES2-EGFP or the derivative ones with inserted cDNA encoding Vim(WT), Vim(S55E), Vim(S54E), or Vim(S38E) and after 18 h were stained with MitoTracker Red and movements of mitochondria were analyzed. Values are mean percentage of movements exceeding 0.2 μm/s±s.e.m.; *n*=number of cells (approximately 500 movements in each cell). *Significantly different from control (*P*<0.01); **no significant difference from control (*P*>0.80).

Indeed, vimentin is a good substrate for numerous protein kinases (Eriksson et al., 2004; Izawa and Inagaki, 2006). PAK1 kinase was shown to phosphorylate vimentin *in vitro* on several sites (Goto et al., 2002). Besides phosphorylation of vimentin by PAK1 was documented in smooth muscle cells on Ser-55 (Tang et al., 2005), in HeLa cells on Ser-72 (Chan et al., 2002). Vimentin phosphorylated on Ser-38 in murine fibroblasts was also found as a result of Rac1 activation (Helfand et al., 2011). Since Ser-55 resides in the region involved in the VIF-mitochondria interaction (Nekrasova et al., 2011), we tested whether phosphorylation of vimentin on this site is involved in Rac1 regulation of mitochondrial motility. For that we expressed the Vim(S55E) mutant in vimentin-null cells mimicking vimentin phosphorylated on Ser-55. Supplementary material Fig. S2I shows that this mutant is able to polymerize into VIF. It can be also seen in Fig. 4 that in contrast to wild type variant that decreases mitochondrial motility this mutant has no such an effect. So, phosphorylation of Ser-55 of vimentin disrupts interaction between VIF and mitochondria. As a control to this observation we expressed two other vimentin mutants: imitating phosphorylation on the other PAK1-specific site Vim(S38E) localized outside the mitochondria-binding region, and Vim(S54E) with mutation of Ser-54 that resides in this region but is not phosphorylated by PAK1 kinase. Both of them formed filaments despite of mutations (supplementary material Fig. S2C,F) but had different influence on mitochondrial motility. While Vim(S38E) decreased mitochondrial motility similarly to wild type VIF, Vim(S54E) did not (Fig. 4). So, phosphorylation of vimentin can cause disruption of its interaction with mitochondria if the modified amino acid is localized in mitochondria-binding region. Thus, altered motility of mitochondria caused by PAK1 kinase could be due to vimentin phosphorylation on Ser-55.

**Fig. 5.**
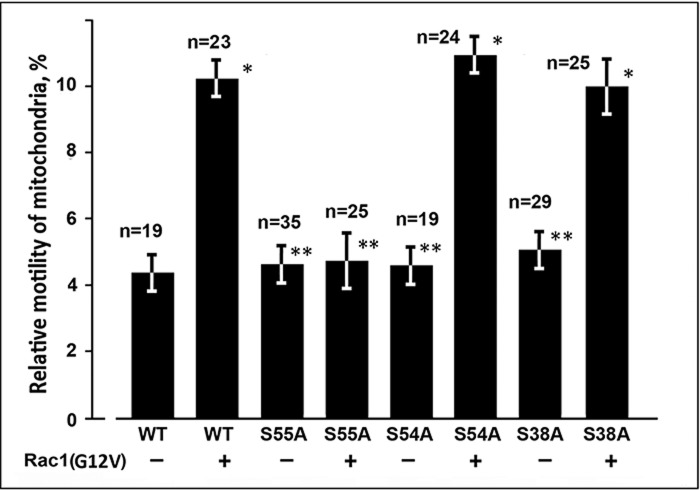
**Phosphorylation of Ser-55 of vimentin is required for Rac1 effect on mitochondria motility.** Cells MFT-16 were transfected either with plasmids derivative of pIRES2-EGFP with inserted cDNAs encoding Vim(WT) (control), Vim(S55A), Vim(S54A), or Vim(S38A) or plasmids derivative of pIRES2-EGFP-Rac1(G12V) with inserted cDNAs encoding Vim(WT), Vim(S55A), Vim(S54A), or Vim(S38A). 18 h after transfection cells were stained with MitoTracker Red and movements of mitochondria were analyzed. Values are mean percentage of movements exceeding 0.2 μm/s±s.e.m.; *n*=number of cells (approximately 500 movements in each cell). *Significantly different from control (*P*<0.01); **no significant difference from control (*P*>0.80).

To further explore the possibility that Rac1 acts through PAK1 kinase activation and phosphorylation of vimentin we restored VIF in vimentin-null cells using mutants Vim(S38A) and Vim(S55A) imitating dephosphorylated forms that cannot be phosphorylated by PAK1 on these amino acids, and Vim(S54A) with substitution of neighboring serine that is not a PAK1 kinase site. For simultaneous expression of a relevant vimentin variant together with Rac1(G12V) in transfected cells we used the plasmids constructed on the base of pIRES2-EGFP in which EGFP cDNA was substituted with that of Rac1(G12V)-EGFP, and the source plasmid with respective vimentin cDNAs were used as a controls . All three vimentin mutants formed VIF networks in transfected cells (supplementary material Fig. S2), and each of them was able to bind mitochondria as follows from the analysis of their motility in transfected cells. Similarly to the wild type variant of vimentin these mutants caused the decreased mitochondrial motility (Fig. 5). However, co-expression of Rac1(G12V)-EGFP suppressed the inhibition of mitochondrial movements only by VIF formed with Vim(S38A) and Vim(S54A) (Fig. 5); the mutation S55A completely blocked the effect of this GTPase. So, these data indicate that Ser-55 is the phosphorylation site in vimentin molecule through which PAK1 kinase, the effector of Rac1 regulates the interaction of VIF with mitochondria.

Since vimentin was reported to contribute in mitochondria morphology (Tang et al., 2008) we sought out whether regulation of the binding of these organelles to VIF could affect their shape. For that we analyzed the morphology and motility of mitochondria in vimentin-null cells and the cells stably transfected with cDNA encoding Vim(S55A) and Vim(S55E). Although the movements of mitochondria in cells expressing Vim(S55A) were significantly suppressed in comparison to those in vimentin-null cells or cells with Vim(S55E) (supplementary material Fig. S4C) the morphology of these organelles was not altered. Supplementary material Fig. S4D shows that the distribution of mitochondria with different circularity that reflects their elongation was similar in these tree cell lines.

### Rac1 and PAK1 decrease the mitochondrial membrane potential

**Fig. 6.**
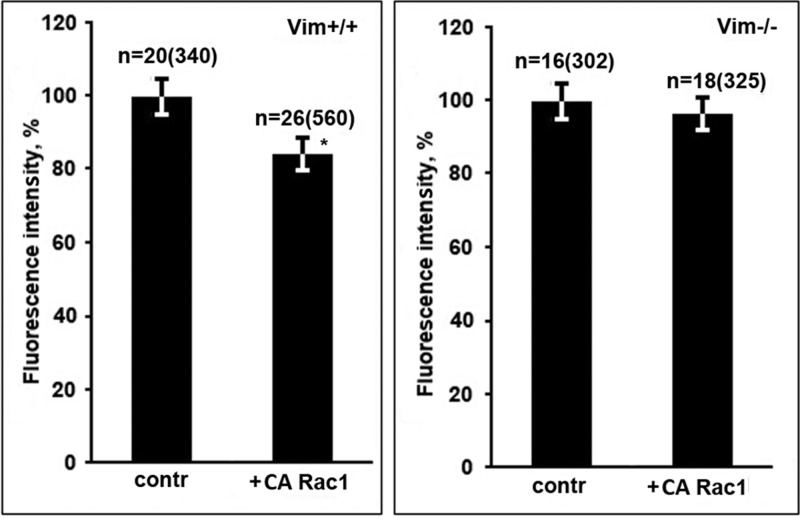
**Activated Rac1 decreases mitochondria potential in vimentin-containing cells.** Cells MFT-6 (Vim+/+) or MFT-16 (Vim−/−) were transfected with plasmid encoding EGFP-Rac1(G12V) or EGFP as a control and after 18 h were stained with TMRM. Values are mean percentage of intensity of fluorescence±s.e.m.; *n*=number of cells (number of mitochondria in brackets). *Significantly different from control (*P*<0.01).

**Fig. 7.**
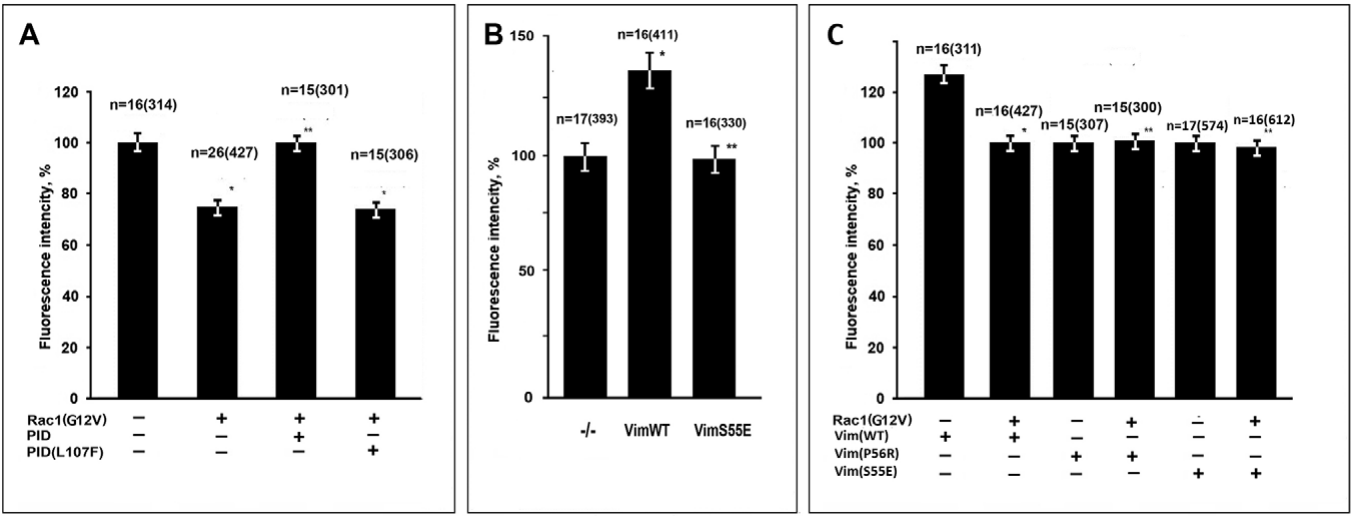
**Rac1 regulates MMP through PAK1 kinase.** (A) Cells MFT-6 were transfected with plasmids encoding RFP-Rac1(G12V), EGFP-PID, and EGFP-PID(L107F). (B) Cells MFT-16 were transfected with plasmids pIRES2-EGFP (−/−) or pIRES2-EGFP-Vim(WT) and pIRES2-EGFP-Vim(S55E). (C) Cells MFT-16 were co-transfected with plasmids encoding Vim(WT), Vim(P56R) or Vim(S55E) with either pEGFP (controls) or pEGFP-Rac1(G12V). 18 h later cells were stained with MitoTracker Deep Red FM. Values are mean percentage of intensity of fluorescence of control±s.e.m.; *n*=number of cells (number of mitochondria in brackets). *Significantly different from control (*P*<0.01); ** no significant difference from respective control (*P*>0.70).

We have shown recently that binding of mitochondria to VIF not only decreases their motility but also increases mitochondrial membrane potential (MMP) (Chernoivanenko et al., 2015). To test whether an activated Rac1 decreases MMP affecting the interaction of mitochondria with VIF we compared the fluorescence intensity of potential-dependent dye TMRM in cells transfected with Rac1(G12V)-GFP or GFP as a control in vimentin-null and vimentin containing fibroblasts. The data in Fig. 6 show that in cells containing VIF (MFT-6) expression of Rac1(G12V)-EGFP decreased the MMP while in vimentin-null cells (MFT-16) it remained on the same level. Thus, Rac1 is involved in regulation of MMP in cells containing VIF. As far as Rac1 controls mitochondrial motility through its effector PAK1 kinase we investigated the possibility that this protein kinase also influences MMP. For that we co-expressed an activated RFP-Rac1 together with EGFP-PID in cells containing VIF [inactive variant EGFP- PID(L107F) was used as a control]. Fig. 7A shows that inhibition of PAK1 kinase by PID completely suppressed the effect of Rac1(G12V) on MMP. To verify that the target of PAK1 kinase in this case is also Ser-55 in vimentin molecule we analyzed the effect of expression of Vim(S55E) on MMP. The data in Fig. 7B show that VIF with mutation mimicking phosphorylated Ser-55 does not increase MMP in contrast to wild type variant. To test the possibility that Rac1 could affect MMP in cells with mutated VIF that by themselves do not alter mitochondria properties we co-expressed pEGFP-Rac1(G12V) with vimentin mutants Vim(P56R) (Chernoivanenko et al., 2015) and Vim(S55E). Fig. 7C shows that activated Rac1 does not decrease MMP in such cells in contrast to cells expressing Vim(WT). So Rac1 controls mitochondrial functions by means of PAK1 kinase which causes the phosphorylation of Ser-55 of vimentin resulting in a disruption of mitochondria-VIF interaction.

### MMP is differently regulated by Rac1 in polarized fibroblasts

**Fig. 8.**
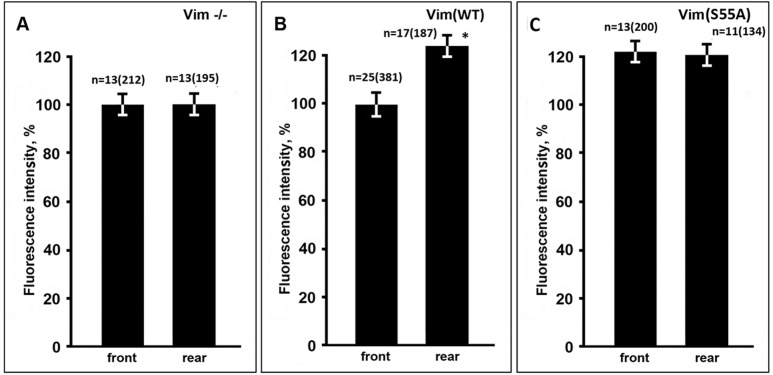
**Mitochondria have a lower MMP at the front of polarized fibroblasts.** Cells MFT-16 (Vim−/−) (A) were transfected with plasmid pIRES2-EGFP-Vim(WT) (B) or pIRES2-EGFP-Vim(S55A) (C) and after 18 h were stained with TMRM. Intensity of fluorescence was measured separately in mitochondria localized at the front half of cellular cytoplasm and at the rear parts of the cells. Values are mean percentage of intensity of fluorescence of mitochondria at the front of non-transfected cells±s.e.m.; *n*=number of cells (number of mitochondria in brackets). *Significantly different from value at the front (*P*<0.01).

It was noticed in earlier studies that mitochondria show wide variation in MMP levels even in the same cell (Collins et al., 2002; Kuznetsov and Margreiter, 2009; Chernoivanenko et al., 2015). Regulatory pathway discovered in the present study could explain such heterogeneity in MMP. Since spatiotemporal distribution of activated Rac1 was implicated in the mechanisms of cell polarization and migration (Machacek et al., 2009) we tested whether uneven activation of this small GTPase in fibroblasts causes polarized distribution of mitochondria according to their level of energy. For that we compared MMP at the front parts of fibroblasts, determined by the pseudopodial activity of the leading edge, with that at the rear parts of the cells. Fig. 8B shows that mean value of the intensity of fluorescence of TMRM of mitochondria localized at the rear parts of the cells was by ∼20% higher than of those at the front. Interestingly, such difference was observed only in cells that expressed wild type VIF while in vimentin-null cells (Fig. 8A) or cells expressing VIF with S55A mutation (Fig. 8C) the potential of mitochondria was equal at the front and the rear parts of fibroblasts. It was also shown that in cells containing Vim(S55A) the level of MMP was increased in both cytoplasm domains (Fig. 8C). So, these data indicate that activated Rac1 at the front of migrating fibroblasts causes the decrease of MMP at the adjoining part of cytoplasm.

## DISCUSSION

Though the importance of an association of mitochondria with IF for their distribution and stability is generally accepted (Uttam et al., 1996; Gilbert et al., 2001; Tolstonog et al., 2001; Capetanaki, 2002), only our recent discoveries (Chernoivanenko et al., 2015) propose that they can be involved in regulation of mitochondrial functions. Indeed, the interaction of VIF with mitochondria that increases MMP could be regarded as a switch that turns on the enhanced ATP production. Considering such possibility the reliable mechanisms that control this interaction could be expected. We have found earlier that small GTPases of Rho family are involved in the interaction of mitochondria with cytoskeleton. So, RhoA and its effector mDia1 were shown to inhibit the mitochondrial movements while Rac1 had an opposite effect (Minin et al., 2006). Acting through different effectors Rac1 and other members of this family control the rearrangements of actin cytoskeleton, microtubules (Etienne-Manneville and Hall, 2002; Wittmann et al., 2003) and VIF (Meriane et al., 2000; Helfand et al., 2011). As it was demonstrated for actin cytoskeleton, the activation of Rac1 in response to receptor stimulation enhances cell spreading and migration by stimulating actin polymerization at the plasma membrane and promoting the formation of lamellipodia or membrane ruffles. In contrast, activation of Rho stimulates cell contractility and adhesion by inducing the assembly of actin stress fibers and focal adhesion complexes. The balance between these two opposing activities is a critical determinant of cellular morphology and migratory behavior.

It was shown in several studies that RhoA is downregulated by Rac1 (van Leeuwen et al., 1997; Sander et al., 1999; Zondag et al., 2000; Nimnual et al., 2003) that results in formation of broad lamellipodia and disruption of contractile stress fibers. So, the increased motility of mitochondria caused by Rac1 activation could be explained by the suppression of RhoA inhibitory effect on mitochondrial movements demonstrated earlier (Minin et al., 2006). However, expression of double mutant Rac1(G12V, Y40H) that caused the inhibition of RhoA which is seen by the disruption of stress fibers in transfected cells (supplementary material Fig. S1B) did not increase the mitochondria motility (Fig. 2E). On the contrary, the other Rac1 double mutant, Rac1(G12V, F37L) which does not alter actin cytoskeleton (supplementary material Fig. S1D) increased mitochondrial motility (Fig. 2E). Thus, the effect of Rac1 is not due to RhoA inhibition.

Another possible explanation of the increase of mitochondrial motility could be the retraction of VIF network from the cellular periphery as a result of Rac1 activation that was shown earlier (Valgeirsdottir et al., 1998; Meriane et al., 2000; Helfand et al., 2011). The motility of mitochondria at the cellular periphery can increase since they have no constraints from VIF. However, the retraction of VIF is not the only reason of mitochondrial “liberation”. The observed partial clustering of mitochondria at the perinuclear area (Fig. 1F) indicates that the bound organelles redistribute together with retracted VIF. Our data clearly demonstrate that double mutant Rac1(G12V, Y40H) capable to induce lamellipodia formation and retraction of VIF does not increase mitochondrial motility (Fig. 2E) while the other mutant, Rac1(G12V, F37L) that does not alter VIF distribution increase mitochondrial motility. Noteworthy, the normal distribution of VIF network in cells expressing Rac1(G12V, F37L) does not impede enhanced mitochondrial motility. Thus, effect of activated Rac1 on mitochondrial behavior is explained not by simple removal of VIF but rather by the disturbance of the interaction of these organelles.

The involvement of PAK1 kinase in the regulation of mitochondrial binding to VIF proposes several possible mechanisms. First, vimentin is a target of this protein kinase (Goto et al., 2002; Chan et al., 2002; Tang et al., 2005; Helfand et al., 2011) and its phosphorylation may provoke the depolymerization of VIF (Eriksson et al., 2004) and/or stimulation of transport of VIF along microtubules (Robert et al., 2014) and as a result the increase of mitochondrial movements. However, our data show that increase of mitochondrial motility is observed in cells with intact VIF network (Figs 1 and 2), though the partial depolymerization of VIF cannot be excluded. Second, the cause of the observed effect on mitochondria could be the phosphorylation of vimentin molecule on the site that is involved in this interaction (Nekrasova et al., 2011). Indeed, the mutations mimicking phosphorylation of vimentin S55E that is located in this site, but not S38E, abrogate binding of mitochondria to VIF (Fig. 4). Besides, the mutant Vim(S55A) that imitates dephosphorylated form that cannot be phosphorylated on Ser-55 blocks the effect of Rac1 (Fig. 5). On the other hand, mutations S54A and S38A do not perturb the regulation of mitochondrial motility by Rac1 (Fig. 5). So, the increase of mitochondrial motility and the decrease of mitochondrial membrane potential occur as a result of the phosphorylation of vimentin on Ser-55. However, it is still to be determined whether PAK1 directly phosphorylates Ser-55 or some other protein kinase activated by PAK1 is responsible for this phosphorylation. Mitotic p34cdc2 kinase that phosphorylates Ser-55 (Chou et al., 1990) could be involved, but it is unknown whether PAK1 kinase regulates its activity.

An establishment of polarized morphology is a prerequisite for cell migration. The involvement of vimentin expression in cell polarization during the epithelial to mesenchymal transition was clearly demonstrated earlier in the Goldman's lab. The assembly of VIF in epithelial cells was sufficient to induce the changes in cell shape and motility typical for fibroblast-like cells (Mendez et al., 2010). Interestingly, lamellipodia formation characteristic for the front edge of polarized fibroblasts requires VIF disassembly and/or retraction caused by Rac1 dependent phosphorylation of Ser-38 of vimentin by PAK1 (Helfand et al., 2011). The concomitant release of VIF-bound mitochondria and decrease of their membrane potential that was shown in this paper provide additional contribution in cell polarization. A lower potential in mitochondria at the front part of a cell and a higher potential at the rear part could be significant for the establishment of different jobs assigned to these cytoplasmic domains. It could be suggested that an enhanced ATP production by highly energized mitochondria at the rear is needed for an effective myosin dependent contraction of a cellular tail. On the other hand the decrease of the MMP at the front might be a prerequisite of a ROS production by mitochondria implicated recently in regulation of cell migration (Wang et al., 2011; Venkova et al., 2014).

## MATERIALS AND METHODS

### Cell culture

Murine cell lines MFT-16 derived from a vimentin knockout mouse and MFT-6 derived from a wild type mouse (Holwell et al., 1997) were kindly provided by Prof. R. Evans (University of Colorado, Denver). MFT-16 cells stably expressing Vim(S55E) or Vim(S55A) were maintained in medium containing 0.5 µg/ml puromycin; antibiotic was omitted during experiments. All cells were maintained in DMEM supplemented with 10% FCS, 100 U/ml penicillin and 100 µg/ml streptomycin. For microscopic observations, cells were plated on coverslips at least 16–20 h before experiments were performed.

### Plasmids and transfection

Plasmid pEGFP-IRES2-Vim that encode EGFP and human vimentin divided by internal ribosome entry site (IRES) was constructed using vector pEGFP-IRES2 (Clontech, USA) by standard procedures and used for restoration of VIF in MFT-16 cells where indicated. Plasmids encoding vimentin mutants Vim(S38E), Vim(S38A), Vim(S54E), Vim(S54A), Vim(S55E), Vim(S55A) were made using inverted PCR followed by blunt end ligation of resulting product (Erster and Liscovitch, 2010) with plasmid pEGFP-IRES2-Vim as a template. The primers used in these procedures were: (S38A) GCTCTGGGCAGCGCG and GTAGGTGCGGGTGGACGT; (S38E) GAGCTGGGCAGCGCG and GTAGGTGCGGGTGGACGT; (S54A) GCTTCCCCGGGCGGC and GGCGTAGAGGCTGCGG; (S54E) GAATCCCCGGGCGGC and GGCGTAGAGGCTGCGG; (S55A) GCCCCGGGCGGCGT and GGCCGAGGCGTAGA; (S54E) GAACCGGGCGGCGT and GGCCGAGGCGTAGA. Plasmid encoding vimentin mutant Vim(P56R) was described earlier (Nekrasova et al., 2011). Plasmids pEGFP-Rac1(G12V, F37L) and pEGFP-Rac1(G12V, Y40H) encoding effector loop mutants of Rac1 and pEGFP-Rac1(G12V), pEGFP-Rac1(T17N) were the gift from Dr Linda Van Aelst (Cold Spring Harbor Laboratory, NY, USA). Plasmids pEGFP-PAK1-PID and pEGFP-PAK1-PID(L107F) encoding auto-inhibitory domain of PAK1 and its inactive version, respectively, were from Dr Ulla G. Knaus (Scripps Research Institute, La Jolla, CA, USA). Plasmid pTag-RFP-C-PA-Rac1 encoding light-activated version of Rac1 was made by reclonning of PA-Rac1(G12V) cDNA from pTriEx-mVen-PA-Rac1(G12V)-N-term2 (kindly provided by Dr Klaus Hahn, University of North Carolina, Chapel Hill, NC, USA) into vector pTag-RFP (Evrogen, Russia). In order to co-express different vimentin variants together with desired Rac1 mutants we made a collection of bi-cistronic plasmids on the basis of pEGFP-IRES2 vector that encode EGFP-labled Rac1 proteins instead of EGFP.

Transfection was performed using *Trans*IT-LT1 Transfection Reagent (Mirus Bio, USA) according to the manufacturer's instructions. 1 µg of DNA was used for a 40 mm dish with 2 ml of medium. Cells were observed 16–20 h after transfection. For live cell analysis of mitochondria motility and potential the flat cells with better distributed mitochondria were usually used.

For retroviral transfection Vim(S55E) and Vim(S55A) cDNAs were inserted into the pBABE-puro (Cell Biolabs, USA) retroviral vector according to the manufacturer's instructions. Briefly, virus was produced by co-infecting equal amounts of the respective pBABE-puro-Vim construct and pCL-Eco helper plasmid (Imgenex, USA) into HEK293 cells using *Trans*IT-LT1 Transfection Reagent. Culture supernatant was collected on the second day post infection and incubated with the target cells for 4–8 h in the presence of 8 μg/ml polybrene (Sigma). Two days after infection, cells were placed under 2 μg/ml puromycin selection. To avoid the effect of the drug resistance mediated by P-glycoprotein during selection 2.2 μM of verapamil was added to the culture medium.

### Immunofluorescence

VIF staining in cell preparations was performed by indirect immunofluorescence using the mouse monoclonal antibody V9 (Chemicon International, Germany) for human vimentin, the polyclonal rabbit antibody RVIM-AT (Avsyuk et al., 1997) for murine vimentin and FITC- or TRITC-labeled goat anti-mouse or goat anti-rabbit (Jackson, USA). Actin cytoskeleton was stained with TRITC-phalloidin (Molecular Probes, USA). Before staining, cells were fixed with 4% formaldehyde in PBS following by permeabilization with 0.1% Triton X-100.

### Live cell imaging

To stain mitochondria for motility assay MitoTracker Red CMXRos or Deep Red FM (Invitrogen, USA) was added to the culture medium at concentration 15 ng/ml and the cells incubated for 30 min before this medium was removed and replaced with fresh medium. For MMP measurements cells were incubated with 25 nM TMRM for 1 h. To avoid the effects of a dye efflux from cells mediated by P-glycoprotein, product of multi drug resistance gene (de Flora et al., 1997), verapamil was added to the incubation mixture to concentration 2.2 μM. Then coverslips were mounted in a chamber filled with DMEM supplemented with 10% FCS. Temperature was maintained at 37±2°C with an Incubator S, (PeCon, Germany). Epi-fluorescence microscopy was carried out with Axiovert 200 M (Zeiss, Germany) equipped with Plan-Apochromat 63×1.4 NA objective. Images were captured with an AxioCam MRm camera (Zeiss) driven by AxioVision 4.6 (Zeiss) software. To minimize phototoxic damage, a 100 W halogen lamp was used as a light source for fluorescent imaging of live cells.

### Quantitative analysis of mitochondrial motility

Image analysis was done as described earlier (Minin et al., 2006) using an open source image analysis software ImageJ (http://rsbweb.nih.gov/ij). Because of the diverse morphology of mitochondria we plotted the coordinates of one end for longer organelles and the center of mass for shorter ones. Mitochondrial motility was expressed in terms of the displacement distances, and velocities. To define fast organelle movements we applied a threshold of 200 nm per second. The values of the relative motility of mitochondria were expressed as mean percentages of fast movements in all recorded displacements±s.e.m. In each experiment individual movements of 20–40 mitochondria were analyzed in each of 10–15 cells. The significance of differences was estimated statistically by the paired-sample Student's *t*-test. Variability of the values calculated for different cells in the samples was analyzed by the same method and was insignificant.

### Computer-assisted quantification of mitochondrial fluorescence

The trans-membrane potential of mitochondria was analyzed with the help of the potential-dependent fluorescent dye TMRM (Molecular Probes). To compare membrane potential of mitochondria in different experimental conditions intensity of TMRM fluorescence was measured using ImageJ software (Chernoivanenko et al., 2015). Average intensity of pixels inside the contours determined for each organelle was measured. Routinely, data from 10–20 cells were collected for each experimental condition and mean values of fluorescence intensity±s.e.m were calculated.
